# Talking points for physicians, patients and caregivers considering Aduhelm® infusion and the accelerated pathway for its approval by the FDA

**DOI:** 10.1186/s13024-021-00490-z

**Published:** 2021-11-04

**Authors:** Sam Gandy, David S. Knopman, Mary Sano

**Affiliations:** 1 Department of Psychiatry, NIA-Designated Mount Sinai Alzheimer’s Disease Research Center, New York, NY 10029 USA; 2 James J Peters VA Medical Center, Bronx, NY 10468 USA; 3 Department of Neurology, Mount Sinai Center for Cognitive Health and NFL Neurological Care, New York, NY 10029 USA; 4 Department of Neurology, Mayo Clinic, Rochester, MN 55905 USA

**Keywords:** Aduhelm® (aducanumab), Amyloid beta, Donanemab, Lecanemab, Gantenerumab

## Commentary

Aduhelm® (aducanumab) is a “biological” new drug recently approved by the United States Food and Drug Administration (FDA) via an “accelerated pathway” mechanism. Aduhelm® is a recombinant antibody protein created by Biogen for the purpose of depleting patients’ brains of amyloid beta (Aβ), a protein that accumulates in the brain as part of the standard neuropathology of Alzheimer’s disease (AD). A distinction worth drawing here is that “clinical dementia due to Alzheimer’s disease” is now recognized as a separate entity from “Alzheimer’s neuropathology” for the simple reason that a patient can have full-blown “Alzheimer’s neuropathology” but remain cognitively intact presumably due to resilience genes and pathways [1, 2].

The use of the “accelerated pathway” enabled the FDA approval of a drug without evidence for meaningful clinical benefit. The “accelerated pathway” provides an FDA imprimatur to a new compound based on demonstration that a drug-related change occurs in a “biomarker” in a patient’s biofluid or as the result of patient’s imaging test and that that biofluid or imaging “biomarker” is expected to be the harbinger of a clinically meaningful benefit. In the case of Aduhelm®, intravenous infusion with the drug can lower a patient’s burden of amyloid “fibril” on positron emission tomography (PET) brain scans [3]. The key points from the two pivotal Phase 3 trials conducted by Biogen were a) that after 18 months of treatment with Aduhelm®, there were not reproducible clinical benefits, and b) the available evidence shows no correlation between the degree of amyloid lowering and the main clinical outcome measures. Thus, when it comes to risk: benefit estimation for Aduhelm®, we are faced with the problem of a zero in the denominator. Because the “accelerated pathway” permits approval based on a biomarker and not a clinically meaningful benefit, risk:benefit ratio is meaningless and incalculable. The “accelerated approval” pathway employed by the FDA “approves a drug for a serious or life-threatening illness that may provide meaningful therapeutic benefit over existing treatments when the drug is shown to influence a surrogate endpoint that is reasonably likely to predict a clinical benefit to patients and there remains some uncertainty about the drug’s clinical benefit”. In this approval process, the FDA relied upon evidence in two clinical trials demonstrating removal of amyloid plaque on imaging and the FDA acknowledges “uncertainty about clinical benefit”. No peer review published data are available from either of the two Phase 3 studies that were submitted for approval. Furthermore, the issue of the safety of Aduhelm® was understated in the FDA approved labeling; even Aduhelm®‘s supporters bemoan this deficiency [4]. Despite data from both studies identifying significant adverse events of various seriousness and multiple limitations and restrictions in the entry criteria of the studies, the approved package insert has nothing listed under “contraindications”.

A 10–1 vote against traditional approval by an FDA Advisory Committee (aka an “FDA AdComm”) in November of 2020 was followed by an FDA “accelerated approval” of Aduhelm® on 07 June 2021 for the treatment of Alzheimer’s disease. The “accelerated approval” pathway had never been entertained by the FDA AdComm and, following the FDA decision, three highly prominent clinician-researcher FDA AdComm members (professors at Harvard, Mayo Clinic (co-author of this Commentary DSK), Washington University in St Louis) resigned in protest.

At least in part because of the nature of the “accelerated approval” pathway used for Aduhelm®, the drug label fails to reflect the design or results associated with the two clinical trials of the drug. While medical centers and professional organizations draft “Consensus Aduhelm ® Best Practices”, we were invited to draft this Commentary to share with the readers of *Molecular Neurodegeneration* some of the key features of Aduhelm ® revealed during public platform and poster presentations at medical and scientific meetings over the past decade as well as points that have been made by patients, families, and clinicians contemplating the use of Aduhelm®.

The major controversy and challenge to clinician prescribers is the massive information gap making it nearly impossible to determine how to inform patients and families about this drug including the lack of guidance and contradictory information:

- Between the FDA AdComm advice and FDA approval
- That Aduhelm®‘s mechanism of action is based on the presence of elevated brain amyloid, but the prescribing information does not require that abnormality to be proved before initiating treatment
- That accelerated approval is based on a surrogate biomarker yet no requirement to measure that biomarker to manage the therapy
- That ignores the mismatch between well-characterized clinical trial populations with multiple exclusions and the broad indication for patients who might be treated with the drug.
- That as of August 30, 2021, there is no peer-reviewed manuscript on the two pivotal trials on which the initial application for approval was based.

While the “accelerated approval” would dictate post-approval continued clinical trials to determine the presence of a meaningful clinical effect, the 9-year period to accomplish this is obviously useless to patients and prescribers now and exceeds the expected lifespan of many of those who are eligible to undergo treatment.

Despite over a decade of research, the reduction in brain amyloid burden has not been consistently associated with meaningful clinical benefit [5, 6]. The current generation of anti-amyloid antibodies is unusually efficient in clearing amyloid, yet there is no evidence for clinical benefit. Indeed, 18 months of treatment with many anti-amyloid agents has shown no change or mild, mostly non-significant worsening. Only in the last few years have antibodies been developed that can effectively remove most if not all detectable amyloid plaques from the brain as detected by amyloid imaging. Of those antibodies utilized at a high enough dose to have this effect, only aducanumab has completed phase III trials. Three other antibodies including donanemab, lecanemab, and gantenerumab have completed phase 2 trials. Published phase 2 data from donanemab showed ~ 33% slowing of cognitive decline on a prespecified endpoint [7]. However, as noted, definitive phase 3 trials have not yet been completed so a clinically meaningful effect remains to be proven.

The cost of Aduhelm® and the prospects for third party reimbursement remain controversial and relevant. The estimated cost for drug alone is US$ 56,000 per year per patient with total costs for infusions, physician fees, etc., estimated to exceed US $100,000 per year per patient.

According to the FDA statistician’s analysis, only patients that carry an *APOE ε4* allele demonstrated an effect on clinical outcomes, and, ironically, those with *APOE ε4* alleles are also more likely to experience brain microhemorrhages ARIA-h (for amyloid-related imaging abnormalities - hemorrhagic) and brain edema (ARIA-e). In trials, subjects were excluded based on the presence of an excess number of brain microbleeds and were dropped from further infusion after the 10th brain bleed. The label written by Biogen and approved by the FDA recommends “radiological stabilization” and implies lifelong monthly infusion.

Critical information about the extent of amyloid lowering by Aduhelm® on an individual basis has not been released by Biogen, so that the issue of duration of Aduhelm® treatment cannot even be discussed at this time. The FDA-approved Aduhelm® label is for Alzheimer’s disease without any requirement to establish the status of the surrogate biomarker.

Our vision of Best Practices for Aduhelm® would include clear communication that the only reliable outcome is reduction in the surrogate biomarker (amyloid fibrils on brain scan or soluble Aβ in cerebrospinal fluid or blood, since Aβ oligomers cannot currently be reliably detected in brain or biological fluids). We would strongly recommend Aβ biomarker determination and *APOE* genotyping with pre-test and post-test counseling and an absence of mild or greater ARIA-H (for amyloid-related imaging abnormalities with hemorrhage) on MR imaging done prior to treatment initiation, as was required in the trials. These are not routinely conducted for diagnosis and reimbursement for many of these will be required to administer the drug safely.

The association of *APOE ε4* with enhanced risk for AD has been known since 1993 but has never been routinely recommended largely because of the inability to protect patients and families from discriminatory practices in employment or insurability [8]. The authors’ personal practices to date include recommendation for *APOE* genotyping in assessing patients for Aduhelm® infusion, and no reasons to reconsider that policy have yet been encountered. That said, the combination of the clinically questionable significance of amyloid-lowering among *APOE ε4* carriers and the enhanced risk of ARIA-h in *APOE ε4* carriers appear, in our estimation, to play important roles in what has so far been a rapidly declining interest among patients and caregivers as they inquire of these authors and are educated about the prospects of Aduhelm® infusion.

## What’s next?

The introduction of Aduhelm® into clinical practice is truly a “dry” run for dementia therapeutics of the future. There are many alternative approaches to AD therapeutics radically different from manipulation of amyloid levels that are slowly being studied through Phase 1 and 2 testing. Despite the claims of some that Aduhelm®‘s approval will spur greater enthusiasm by the pharmaceutical industry, we believe that the enthusiasm is already there for finding therapeutics that actually have clinical benefits. That said, the accelerated approval of Aduhelm® has induced Lilly (donanemab), Eisai-Biogen (lecanemab) and probably Roche (gantenerumab) to file under a similar “accelerated approval” pathway with approval likely merely based on amyloid lowering. If any of these other three agents were to show a consistent pattern of clinical benefit, however small, use of Aduhelm® would be moot. The absence of currently available published peer-reviewed phase 3 data for all three anti-amyloid antibodies complicates our ability to take supportive positions on any of the three antibodies. The company-presented or online-published data for aducanumab and lecanemab [8] do not obviously trend toward the likelihood that either will yield a clinically meaningful benefit [6]. A phase 2 trial of Donanemab suggests achievement of meaningful benefit on a prespecified endpoint, but that benefit fails to correlate with changes in amyloid biomarker status^7^, a point that undermines confidence in this invocation by the FDA of its accelerated approval pathway. We encourage greater transparency from the FDA to understand the data to support the accelerated approval and how that informs the physician and patient facing recommendations.

## Reimbursement

On 12 July 2021, the Center for Medicare and Medicaid Services (CMS) announced that its comment period on Aduhelm® would commence and be open until April 2022. The expectation is that most decision on 3rd party reimbursement for Aduhelm® will be forthcoming until CMS renders its decision. The typical pattern is that 3rd party payers follow the lead of CMS in determining reimbursement. At present, there is no evidence of widespread adoption of reimbursement.

## Statement of intent

These talking points were drafted as an intended service to the private patients of the authors, and no endorsement by the authors’ employer(s) is/are implied. The views represented here are entirely the personal views of the authors.

## Data Availability

N/A.

## Abbreviations

FDA: United States Food and Drug Administration
AdComm: Advisory Committee
Amyloid beta: Aβ
Apolipoprotein E epsilon 4: *APOE* ε4
ARIA-h: Amyloid-related imaging abnormality-hemorrhagic
ARIA-e: Amyloid-related imaging abnormality-edema
CMS: Center for Medicare and Medicaid Services
